# Supplementary material for: Narratives of most significant change to explore experiences of caregivers in a caregiver-young adolescent sexual and reproductive health communication intervention in rural south-western Uganda
Source: PLoS One. 2023 May 31;18(5):e0286319. doi: 10.1371/journal.pone.0286319 (PMC10231775; doi:10.1371/journal.pone.0286319)
Supplement: S1 File — (DOCX) [file pone.0286319.s002.docx]

**Codebook for MSC stories**

| **No.** | **Domains of change** | **Sub theme** | **Quote** | **Comment** | **Analytical Notes** |
| --- | --- | --- | --- | --- | --- |
| **Story 1** | Parenting skills | Gender differences | Before, I never cared so much about my child, or even never cared that she had become an adolescent. I even didn’t understand much about adolescence. But ever since the intervention started, I learned that there’s a lot a parent contributes to the growth of a child, and when I understood this, I started paying attention to my daughters especially this one who is ten years. She never cared about herself because as a parent, I didn’t teach her how to do it. She never groomed herself and I also never minded a lot. But after the intervention, I taught her why she needs to start grooming herself. I taught her what we had learned about how to keep herself clean and if she ever needs anything, she should ask me and not anybody else. | Parenting and supporting children of a different sex. Now he knows the changes occurring in his daughter as a father. | Paying more attention to the development of the child into an adolescent. Understanding that the parent plays a significant role I helping the child navigate these changes. Being able to discuss with his daughter about body hygiene. |
|  |  | Parenting styles | Before then, my children used to walk around the neighborhood any how without my permission and I wouldn’t even care where they have gone. After the intervention, I took it upon myself to talk to them and now before they go anywhere, they ask for permission or inform me where they are going. | Parents has changed from a neglectful parent and is more involved, paying attention to where his children go. | The caregiver is more involved in the child's life by being keen on the child's movements. However through communication with the child, he is able to monitor the child's movements |
|  |  | Parent child relationships | From these conservations, my wife and I have become friends with them. I also learned that when you talk to your children, they learn good manners from you and you have to set an example for them. I also learned that if you don’t give your children the things they want; they will get them from somewhere else. As adolescents, they are easily persuaded by boys who give them small things; but if you give your children what they want, they won’t be easily persuaded by boys. | Positive and warm relationships between father and his daughter as well as other children in the household | Becoming friends with their children, talking to them and instilling good behavior. Making effort to provide for their children to avoid children seeking them from other transactional relationships |
|  |  | Open and positive communication | From intervention, I learned to listen to what my children are saying and not that everything thing I say is always right. Sometimes I would even go through their mother so that she talks to them. But now, I became friends with my children and so they can’t hide anything from me. | The parent openly discusses with is children and also listens to their opinions | Listening to the child's opinions, avoiding third party communication |
|  | Parent-child SRH communication | Parent communicates on Body hygiene with daughter | Before, I never cared so much about my child, or even never cared that she had become an adolescent. I even didn’t understand much about adolescence. But ever since the intervention started, I learned that there’s a lot a parent contributes to the growth of a child, and when I understood this, I started paying attention to my daughters especially this one who is ten years. She never cared about herself because as a parent, I didn’t teach her how to do it. She never groomed herself and I also never minded a lot. But after the intervention, I taught her why she needs to start grooming herself. I taught her what we had learned about how to keep herself clean and if she ever needs anything, she should ask me and not anybody else. | Parent acknowledges that before the training, he never paid attention to his daughter hygiene. After the training he can talk about it. | Parent acknowledges that there is a lot a parent can contribute to the growth of their child. He now pays attention to his daughter especially on body hygiene |
|  |  | Menstruation | Recently this daughter of mine got into her first periods and her mother wasn’t around but she came and told me. She came running and told me that *“while I was urinating, I urinated blood.”* I quickly went and bought for her pads. This all happened because I had talked to her about her menstruation just like we were taught during the intervention. | Despite the parent not being present, he is able to discuss and support the daughter and talk to her about her menstruation even in the absence of the mother. |  |
|  |  | General SRH | After the intervention I talked to my children about sexual and reproductive health. My 10-year-old daughter has three older sisters. They listen to what I say and we have become close because now we openly talk to them about sexual and reproductive health. | Discussions on SRH are open compared to the situation before | Parents can compare the situation with their older children where they felt uncomfortable discussing SRH with their children. But they can now do it as a result of the training |
|  | Knowledge and attitude on SRH | Increased awareness on puberty | Before, I never cared so much about my child, or even never cared that she had become an adolescent. I even didn’t understand much about adolescence. But ever since the intervention started, I learned that there’s a lot a parent contributes to the growth of a child, and when I understood this, I started paying attention to my daughters especially this one who is ten years. She never cared about herself because as a parent, I didn’t teach her how to do it. She never groomed herself and I also never minded a lot. But after the intervention, I taught her why she needs to start grooming herself. I taught her what we had learned about how to keep herself clean and if she ever needs anything, she should ask me and not anybody else. | Parent acknowledges through training, they were able to understand the significant role of a parent in navigating growth changes in adolescents | SRH discussion experiences on body hygiene of the daughter |
|  | Personal and Family life | couple relations | Also, my wife and I never used to plan together but after the intervention, I have put into practice some of the things taught. We now work together and plan together. | There is a warmer relationship between the participant and his partner |  |
|  | Community level changes |  |  |  |  |
| **Story 2** | Parenting skills | Positive parenting | I learned that when you become soft and listen to your children they will become your friends and also give you time. I have become soft to my children, I have befriended my children and learned to treat my children differently. For example, for the older children, I first tell them stories, ask them how their day was, what they saw, where they were and who they interacted with before I can start communicating with them about sexual health issues. | There is a warmer relationship between the parent and the child that allows open and free discussions on SRH. | Becoming affectionate towards children, employing listening skills, and giving them time |
|  | Parent-child SRH communication | Increase comfort in discussing SRH with child | Before the intervention I used to fear talking to my children about Sexual and Reproductive health, or adolescence. My children would fear too talk to me because I wasn’t close to them. But after the intervention I started talking to them, we became friends. Before then, I wouldn’t even have conversations with my children because they used to fear me, they couldn’t even tell me anything that could have happened to them but now they come and tell me everything like what they saw, where they were or what happened to them where they were. | Parent acknowledges that they are more comfortable discussing SRH with their children compared to what the situation was before | There is less fear among caregivers to discuss SRH matters with children and children are less fearful approaching their caregiver on SRH matters. |
|  |  |  | Every time I would leave the intervention training, I would go home and teach my children what we had learned that day. I taught my daughters to always come to me in case they want anything and they learned it. Before then, they would tell other people; but now, they come to me even when they are in their periods and need pads or knickers. They now tell me who their friends are or those they no longer want to be friends with. I ask why they don’t want to be friends with people anymore and they explain to me. I also advise them accordingly. | The child can openly approach the parent on different issues including their SRH because of the warmer relationship between the caregiver and the child | The children depended on other people for support but now they can approach their caregivers on different matters because there is now a friendly relationship with their caregiver |
| **Story 3** | Parenting skills | Positive Parenting | The first thing I learned from the intervention was to become close to my children and even become friends with them. One thing I have seen and experienced is that if you become friends with a child, they also become friends with you and even share with you some of their concerns. I learned not to shout at my children but before then, I was quarrelsome and tough on my children. I would beat up my children before even asking why they were late from gathering firewood or fetching water. But now, I first ask why they are late or if anything could have happened on their way back home. I also tell them that its bad to come back home late or advise them if anything bad could have happened. | The relationship between the parent and the adolescent is warmer and friendlier | Being friends with their children and being close to them. Acknowledging that when you become friends, they also become friends with you and are bale to chare their concerns. Avoiding punitive approaches like shouting and corporal punishment. Negotiating good behavior by understanding the drivers of the unacceptable behavior. |
|  |  |  | I always make some popcorns at home and tell them *“whoever comes back home early will eat the popcorn and those that will come late will find when the popcorn is finished.”* This makes them come back home early. Also, because I am close with my children, they now tell me the challenges they face. I also tell them that *“you see you have matured and your breasts have developed, so if you allow a boy to touch your breasts or sleep with him, you will become pregnant, get HIV and die.”* | Providing social rewards to children | social rewards to encourage good behavior such as coming back home in time. Also reports of SRH communication by relating the physical changes in the body to SRH risks |
|  | Parent-child SRH communication |  | I see this intervention has helped us a lot in becoming close to our children. Before, we would leave this responsibility for teaching the children about SRH for teachers at school. Teachers at school don’t have time to teach the children and the children fear their teachers, so they can’t ask serious questions. Recently I was telling my adolescent daughter that her breasts have grown and no one should touch them or tell her that she’s beautiful and she believes. I told her I am the only person who should tell her that she’s beautiful and if anyone ever tries to touch her breasts, she should immediately come and report to me. | Acknowledging their role as SRH communication and communication of SRH with their children |  |
| **Story 4** | Parenting skills | change in parenting styles | Before then, I was very authoritative and would order my grandchildren around. But now when I am talking to them I smile even if they have done something wrong. Actually after the intervention, I also taught their grandmother because she would shout angrily at our grandchildren and even sometimes tell them that they want to be like their mother who left them alone. I taught her and told her that it’s not our grandchildren’s fault that their mother left. I had to teach her that we are now their parents and so she also changed the way she talked to them. Our grandchildren are now happier and free with us. One of our grandchildren who is a boy used to fear his grandmother a lot but when I taught her what I had learned from the intervention, she has changed and now she shows them love and the boy no longer fears her | From authoritarian to authoritative parent |  |
|  | Parent-child SRH communication | Increased comfort in discussion SRH | This intervention made me happy and taught me very many things. I will give you an example; one of my grandchildren who is a girl was never my friend, but from the intervention we learned that we should be our children’s friends and now I am close to her. I would like to give you a living example, my granddaughter who is 11 years recently got her first period, but she came and told me. She asked me and said “*Shwenkuru* (Grandfather), I saw blood while I was urinating.” I calmed her down and told her not to worry; I quickly went and bought her pads. I learned this from the intervention; I told my grandchildren that “if anything ever happens to you, you should quickly come and tell me.” | The parent acknowledges that they improved friendship between her and the adolescent has allowed for open discussions to take place on SRH |  |
|  | Knowledge and attitude on SRH |  | Also recently, my youngest granddaughter who is 6 years was recently from the well, she came running and told me that there was someone who tried to rape her while she was fetching water. This happened because I taught all of them that if anything ever happens to you, you should come and report to me and not any other person. | Children can comfortably report to their caregivers any incidents of violence |  |
|  | Personal and Family life |  | My children are now clean and enjoy cleaning our home. We had visitors at home just the other day and when I told them that we were expecting visitors, they woke up early and started cleaning because I had taught them that we have to clean and even welcome visitors. My grandchildren now go to church every Sunday for prayers. Personally, I never used to pray religiously but after the intervention and learning that I have to be an example, I go to church every Sunday, unless otherwise. | Children full participate on domestic activities |  |
| **Story five** | Parenting skills |  | I used to shout at my children, but when I was taught, it calmed me down and now, I talk to them calmly. Even sometimes my children would hide or not talk to me because I used to shout at them but now the children talk to me because I don’t shout at them and they are friendly with me. The truth is that we were ignorant about some of these things because we were also raised this way. But after the intervention, we learned and now we have improved the way we relate with our children. | Friendly relationship between the child and the caregiver | Parents report a change in the way they relate with their children. By being polite when communicating with them- talking softly and calmly |
|  |  |  | I learned how we are supposed to welcome people, greet them give them something to eat when they come. I also learned how to talk to my children about sexual and reproductive health (SRH) and how to become friends with them. So to start talking to my children, I first pray for them, I ask them questions like what could be bothering them and that’s how we start the conversation. When I am talking to them, I talk them softly and calmly. With this approach, they have become my friends, they even talk to me. Now when I send them to the market, they respond very first without even complaining. |  |  |
| **Story six** | Parent-child SRH communication | Increased comfort of SRH communication | The change that has happened in my family is that I used to fear talking to my children specifically about SRH (Sexual and reproductive health) issues. But after we had been taught, I had to become friends with my children and talked to them about SRH. | due to friendly relationships | friendlier relationships reduce fear of discussing SRH |
|  |  |  | . For example, before the corona outbreak, when children were still going to school, my son got issues at school but because he used to fear us, he kept quiet and never told anyone. It’s until I went to his school that I discovered that he had issues. If I had been friends with my son, he would have talked to me freely but he kept quiet because he feared me. | Reports change in comfortability of talk to his child |  |
|  | Parenting skills | Children no longer fear their father | Before then, my children were disrespectful and used to fear me. When I talked to my children, they now no longer fear me and are respectful to me and their mother. The techniques I use to talk to my children is using time when we are all seated together like having lunch or supper. Then I use the notes we were given during the intervention and I tell them topics related to SRH or any other we were taught. |  | Adopting techniques to create comfortable situations for having conversation |
|  |  | Change in parenting styles | As a parent, I have also changed because I used to shout at my children or beat them without asking or understanding why they have done what they have done. But I changed after the intervention and learning about becoming friends with my children. I used to get angry at my children every time they made a mistake; but after the intervention I learned that you have to talk nicely to your children even if they have made mistakes. I taught my children not to walk around the village without my permission, I also taught them that it is important to groom and be clean all the time. |  | Friendly relationship with children |
|  |  | Parenting Roles | I also learned not to just spend money like buying alcohol while my family doesn’t have food. I also learned how to live in harmony with people without fighting with them. |  |  |
| **Story seven** | Parenting skills |  | There were changes for sure. Before this intervention, our children wouldn’t give me time nor listen to whatever I would tell them. As a parent, I would get irritated, find myself annoyed, get stressed out and speak with anger. But now, I see change because when I talk to my child as I was taught, they listen to me. Now, I don’t have to quarrel and the stress has reduced. So really, there was change. If I send my child somewhere, she now comes back in time. |  | changes in the method of communication and applying more listening |
|  |  |  | The other change that happened to my child and in my home is that wherever I would send her to the shop and she never used to bring back the change. But now when I send her to the shop, she immediately brings what I have sent her and brings back the change. She also goes ahead to tell me what and who she has met on the way and what that person told her, which wasn’t the case before. This happened because the information I got from the sessions taught was put into practice. I became close to my child, I talk to her, I advise her, and yes she became open shared with me her basic needs which she used not do previously. So this intervention was very good because it taught me to befriend my child and now see the changes, I have come to notice on a child which are really good and positive changes. |  | The children are more open to their caregivers due to the calmer relationships |
|  |  |  | During the training we were taught about how to guide a child as he nears the adolescence stage. I had a girl of 14 years and out of the blue she started to change. For example, when I would send her for water, she would delay there so much and by the time she would come back, I would already be annoyed with her. But when we started this training and learnt how you can guide your child, I learnt that when a girl child is in her adolescence, you are supposed to call her and talk to her calmly, like asking her what delayed her at the well yet she was supposed to bring the water earlier. Since then I started handling her slowly by slowly and as I talk, my girl is now doing well. Now I no longer use a loud voice when am talking to her, so she listens and understands when am talking to her. |  | Employing a calmer tone in communicating and listening to the child to allow space for encouraging positive behavior |
| **Story eight** | Parenting skills |  | I think my girl has changed because I befriended her and now, we are very good friends. I no longer hide anything from her that can put her life in danger. I also openly tell her what has been spoiling her and because of not hiding anything from her, am noticing a serious change in her. |  | There is more openness in the relationship between the child and their caregiver |
|  |  |  | There are some things that we already know but this training also helped. For example, I used not to be close to my children but after the training, I learnt to befriend my children which is vital. |  |  |
|  | SRH communication |  | Concerning adolescence, I told her that *“my girl you’re now growing, so when you start menstruating, please don’t fear me, instead tell me and I guide you on what to do.”* When your girl child starts her menstruation periods, you’re supposed to tell her how she is supposed to clean her body parts and you also advise her to avoid boys’ peer groups. What I think is that when my girl is in her menstrual periods, she should keep herself very clean because she can easily smell for people, I also told her to keep her arm pits tidy by shaving them in case they develop hair. |  | Assumption on what is expected of her child. Makes pledge to her child to guide her child when she starts menstruation |
|  |  | Comfortable discussions | Before this training I used to feel shy in front of my daughter but nowadays, I feel very free and very okay to talk to my girl about her body changes. Good enough, she was even still young. My daughter is now a changed girl; whenever I talk to her, she responds positively, she even quit the groups that used to delay her at the well. I think my advice has changed my daughter positively. |  | parent can comfortably discuss with her child about the bodily changes. Appreciates the change in her daughters behavior due to communication |
|  | Knowledge and attitude on SRH |  | Apart from this training, when you have a child who is growing, you can advise him/her to go to hospital and get condoms so that when controlling the body fails, condoms can be used, this training further taught us about going for checkups to know our health statuses. |  | caregiver shares her own personal experience on cultural differences and how these influenced her marital relationship |
|  | Personal and Family life |  | Personally, there are things that I never knew that I learnt from this training. For example, I grew up not knowing that when you get married, you are supposed to respect all people you find in your husband’s home, asking about what you don’t know, for example your husband’s taboos. A case in point the family am married to doesn’t take grasshoppers but back at my home we used to eat them so when I reached here, I went to the market and bought them because I loved them so much but when I reached here, my husband told me that “here, we don’t eat grasshoppers.” So how do you think I played it! My husband advised me not to eat them and he pardoned me for bringing them home because I never knew but since then I gave up on them. |  | caregiver shares her own personal experience on cultural differences and how these influenced her marital relationship |
|  | Community level changes |  | In our area it’s not that I only talk to my child alone but also if I find my neighbor’s child in a bad act or bad group, I can tell her that “my child, what we are being trained concerns you so this is how you should behave.” |  |  |
| **Story 9** | Parenting skills |  | This training helped so much in my family and especially my children and my husband. You know for us village people, what is important is having children and leave them there to whom it may concern. But when I started this training, I came to know the importance of a child, how to look after your child and having time for your children and this helped me to befriend the child and we talked at length which had never happened before. This enabled me to know her problems, her likes and dislikes. I went ahead and shared with my husband and after every session, I would tell him what we had studied and he also liked it. He also engaged the boys and they talked. So nowadays, there is effective communication between us and our children in general there was a very big positive change noticed in my family. |  | Acknowledging how neglectful they have been but after the training the understand their role as the caregiver of the child and creating time for the child |
|  | Personal and Family life |  | As children grow, married couples change their ways. The way I behave now is different form the way I behaved with my husband when my children were still young. In this area, some families do not have enough food, no stable income which makes one unable to hire land for farming and this goes on to affect the children’s studies it all rotates on unstable income. |  | structural challenges affecting parent relationships |
|  | Community level changes |  |  |  |  |
|  | Intervention Delivery |  | What caused that was attending the training because the workshop opened my ears and eyes and after I put into practice what I had studied. Concerning the government programs, we as villagers normally don’t respond to those callings even at churches. Sometimes, we fail to go there. But for this training, we were well mobilized and just given one day in a week which was convenient being the major reason we attended and we loved it. Even when we went for the training, we found there very good teachers who taught very well indeed. This training was very fruitful. |  | Intervention delivery went on well compared to previous intervention in the community |
| **Story 10** | Parenting skills |  | The change that I got I was too busy with the worldly jobs and never had time for my children. I valued my work a lot but when I started attending the training, I developed a very strong relationship with my children. Surely this training helped me learn a lot that I never knew about my children! In short, I never knew anything about the stories of my children. I was a rigid and principled parent and too tough. Actually, my children used to fear me. But after this training, I developed time talk to my children and now they are my friends. This has brought a very big change in my family. I even heard my children asking themselves *“what happened to mum? These days as she has too much time for us.”* I have noticed a big change between me and my children. Now that we spend more time together at home and in the garden, I often talk to them and even narrate to them what I learnt in the training. For example, I don’t allow my children to loiter around. Even now that am not there, I left them with what to do so as to keep them busy. I learnt that from this training and so it has helped to them moving around during this corona break. |  | Setting in priory to build relationship with children |
|  |  |  | I have my elder daughter, I never noticed it when she started having her menstrual periods. But now, the younger sister, because of this friendship we developed, when her time came, she told me this. I noticed that as a big very change, meaning that the elder sister never told me because I never gave her time. |  | the change in relationship has enabled children to easily approach their caregivers on some SRH matters |
|  | SRH communication |  | As a parent I never knew that a girl of 12years can go into her menstrual periods or become pregnant. I always looked at her as a child but after attending the training, we now seat together and I tell them everything even if one is 13years I tell her that “boys can impregnate you.” I go ahead to tell her about her body changes, I even have a boy of 15years. So, when I notice that he has also started feeling so high, I talk to him and warn him about other girls. Because of this training especially in our cell, we have not had problems of children being raped, defilement among others due to the redundancy caused by corona and this was as a result of the study we had with a team from Mbarara University. |  |  |
|  | Knowledge and attitude on SRH |  | As a parent I never knew that a girl of 12years can go into her menstrual periods or become pregnant. I always looked at her as a child but after attending the training, we now seat together and I tell them everything even if one is 13years I tell her that “boys can impregnate you.” I go ahead to tell her about her body changes, I even have a boy of 15years. So, when I notice that he has also started feeling so high, I talk to him and warn him about other girls. Because of this training especially in our cell, we have not had problems of children being raped, defilement among others due to the redundancy caused by corona and this was as a result of the study we had with a team from Mbarara University. |  |  |
| **Story 11** | Parenting skills |  | I loved myself and actually I missed for only two days. This study has helped all my two children. I normally talk rudely to my children but of late I talk politely to them. This study has really made me love my children. We sit and talk together and we love each other. I have a boy and a girl; I tell them not to sleep with each other because they are brother and sister. They listened to me. |  | Being able to form a friendly relationship with his son and talk and can now talk to them. |
|  |  |  | Yes, you people really taught us and we love ourselves now. My child stays here with her grandmother. I stay down there. I used to ignore her and stay with my son but currently, I have no problem with them all, we are ok. Things are now alright. |  |  |
|  |  |  |  |  |  |
| Story 12 | Parenting skills |  |  |  |  |
|  | Intervention Delivery |  |  |  |  |
|  |  |  |  |  |  |
| **Story 13** | Parenting skills |  | This training has helped me to improve hygiene in my home even the way I handle my children has changed. I no longer beat them like I used to do. Now we seat and talk and try to solve issues. Even when I see a neighbor’s child in wrong, I try to guide her. Here I have older children and I learnt to give them their own bedroom so that they don’t hear me and their dad at night making love. Before then, I used to let them sleep near our marital bed which I realized that is not good |  |  |
| **Story 14** | Parenting skills |  | This training helped me a lot because nowadays, I talk to my children and they also tell me what is bothering them which was not the case before. What is more interesting is that every child always wants to tell you something which makes me so happy. So the training changed my family so much because even my husband loved whatever I would share with him after the training. I also noticed that he is now a changed man. |  | There is open communication between the caregiver and the child |
|  |  |  | I used to be very harsh to my children; in case anyone wanted to tell me what had happened to her but now I learnt to listen to my children and it’s working. For example, since lockdown they are the ones grazing animals but now days, one comes and tells me that *“a certain boy was telling me this and that.”* This used not to be the case. I realized that my talk with them has helped a lot. |  | The parent reports no longer being harsh to her children and handling them in a softer manner. Now the children can openly share some of their personal experiences in the community with the caregivers. |
|  | Knowledge and attitude on SRH |  | . I loved it because it taught me what I never knew. For example, a parent should seat and have a talk with his children. I used to look at my children as very young with little knowledge and I saw no need to discuss with them anything. I learnt from the training and I loved it so much. |  | Parents had the assumption that YA are young and ignorant |
|  | Personal and Family life |  | This training helped me a lot because nowadays, I talk to my children and they also tell me what is bothering them which was not the case before. What is more interesting is that every child always wants to tell you something which makes me so happy. So the training changed my family so much because even my husband loved whatever I would share with him after the training. I also noticed that he is now a changed man. |  | An improvement in the relationship between the participant and her spouse. |
|  | Community level changes |  | This change has affected the whole village; for example, I have my fruits down there. I think you can look at them; avocados, oranges, mangoes, guavas and sugarcane. I had children of my in-laws here who used to go there as destroy them but since the training, I have realized a serious change because there is even a child I caught from my garden of fruits recently and when I took him to the mother, she tried to advise him. Since then I have not seen anyone there, so sincerely this training helped a lot. |  | Parenting…. |
| **Story 15** | Parenting skills |  | This training was very vital to me personally. I used to fear talking to my children but since we had this training, I find it easy and helpful to talk to my children. It’s from this training that started befriending my children and now we are close to one another. I had a belief that the older girls would learn everything from a secondary school, so for them, it’s too late. But for the case of the young ones, we are moving together and am ready to guide them accordingly. I am now teaching them how a girl walks, talks, respects parents and relatives, being welcoming, keeping her personal hygiene and above all being God fearing because it’s the beginning of wisdom. I am even teaching them how to be patient. For example, I am a farmer and their dad is a builder. Therefore, our money doesn’t come in at the same time meaning that they have to be patient with us. |  | Socializing children in a gendered way teaching girls to be submissive |
|  | SRH communication  Knowledge and attitude on SRH |  | Now they are becoming twelve years so I was asking them why they no longer bathe in my presence and they laughed. I advised them not to again take a bath with their brother because they have developed big breasts, armpits, pubic hair which their brother doesn’t have. Before the workshop, my girls used to sun dry their knickers outside but now I taught them to keep hanging them inside their bedrooms in fact I pray to God to bless you because this training that you brought for us has made me create a very strong relationship between me and my children; thank you so much |  | Gendered SRH communication. However there is also misrepresentation of what was taught in the training sessions |
|  |  |  |  |  |  |
|  | Personal and Family life |  | I even learnt being patient because before when I would ask for money from my husband and he says that he doesn’t have it, I would immediately start quarreling that he spent all the money on alcohol and his other wives but now am very patient with him. |  | Improved relationship of the participant and her partner |
| Story 16 | Parenting skills |  | During the training, we were taught to befriend our children by bringing them closer so that they feel free to share with us their challenges. This would help us find ways of helping them. This turned out to be fruitful because as we talk, I am very free with my children and share at ease. They no longer fear me; both the old and the young because I have a girl of 13 years, another one has 8years and an old boy of 18years, I also don’t fear them. |  |  |
|  |  |  | At the start of the training, I even would feel shy and wonder how I would mention some difficult terms in front of my children. But now, all is well because we are very close to each other. This is because of the tricks we were taught to use to approach our children. Now they tell me all their problems without fear and I also guide them accordingly. |  | Increased comfortability of being able to hold discussions with their children without fear |
|  | Community level changes |  | By the way this training has helped the whole village because a bigger number attended the training. So in case you meet the neighbor’s child misbehaving, you can guide him and the neighbor takes it with a good heart which was not the case before the training. Generally, since we trained, there is a very big positive change in this village on our children especially during this Covid time. We have not had any pregnant girl around and all this is as a result of training. |  | Co-parenting at the community level, parents supporting each other in parenting their children |
| **Story 17** | Parenting skills |  | But ever since we were trained, now I see peace in my family. For the case of the children, I would only talk with a stick and in case of anything, I would beat them. But now, I no longer beat them, I talk to them well and they listen and respond. In fact, now there is no noise at home like it used to be. |  |  |
|  |  |  | I have taught my children, praying to God, greeting, and I have befriended them and now they feel free to share with me in case of any challenge. My elder son is a step son but now he is free with me. He has started sharing with me his challenges something he used not to do before. This training has surely brought happiness in my family. I even taught him how to keep his personal hygiene and he also told me how other children were telling him that am not his real mother but surely I have befriended him and we are very close. This has made me happy. Even when my biological children abuse him, he tells me and I advise them. Generally, a very good relationship is being built in my family as a result of this training. |  | Positive relationship between caregivers and their non-biological children |
|  | Personal and Family life |  | Originally when I would send my husband for something and he doesn’t come with it, we would quarrel but now I adjusted and was taught that money at one time can be scarce and what is important is being humble. |  |  |
|  | Community level changes |  | This training has greatly improved my village because one time I beat a child of my neighbor and she reported me to the local councils that her children are not supposed to be beaten by any other person. But now days, you punish a child of a neighbor and she takes it with a good heart which is as a result of the training we had. The changes that am noticing in this area is that for example old boys around used to move around smoking, fighting, taking alcohol and running after our girls which improved greatly after the training. This has helped our village so much. |  |  |
|  |  |  | All these changes are credited to the training we from the Mbarara University team and nothing else. For example, in our village it was no longer possible to go to a neighbor and ask for flour, salt, drinking water. Children have started shaving hair very decently not like how they used to shave funny styles that are not decent. |  |  |
| **Story 18** | Parenting skills |  | I loved the sessions because they taught us on how to get closer to our children, befriending them and raising them with good manners. We didn’t know that as a parent, if a child is your friend, they will no longer fear you and in case of any challenges faced, the child will come and tell you. I have two children 9 and 13yrs and I make sure I keep them closer to me. For example, when I ask why they were not able to fetch water as agreed, the tell me this and this happened. |  |  |
|  |  |  | As a trick of conversing with my children, I invited them to the house and told them life has changed and that I am now a better parent. I started by asking how home was, if saucepans were washed, goats grazed and told them I was now saved and didn’t want to shout at them anymore. The children made fun asking if I had got saved or still a catholic. I told them “no, I haven’t gone to charismatic but saved from talking rudely to you.” I told them to always love me and will always give whoever loves me a sweet to eat. I keep bringing them sweets when am away from home as a pass to getting information on who fought with the other. I advise them not to fight. I also tell them that whoever weeds the garden, will be my friend. So they all compete to do better that the other. However, Covid 19 is what disturbed us, we would be far by now. |  |  |
|  | SRH communication |  | When my children got closer to me, I taught them what I learned from the study sessions especially my girl who is now 13 years old. I told her to hurry and tell me when time comes and she sees blood from her body (menstruation). She asked me why and I told her to tell me and I would tell her what it means then instruct her on what to do next. She took it with a good heart because when I ask her if she has seen any changes, she positively responds. |  | There is willingness to discuss SRH but the still not so open |
|  | Knowledge and attitude on SRH |  | My most interesting topic and what I learned the most is a case where a young girl who stays with the grandmother, or mother or relative is sent for something but is raped along the way. We always knew that the first thing is to run to the LC1 and police and report the case. But instead found out that rushing this child to the hospital would save her from many things like pregnancy, HIV/AIDS. I loved this so much, because we didn’t know about it as village people. |  |  |
|  | Personal and Family life |  | When you study, there’s always something new you will learn and will change your ways of living. You change to being smart and can’t just be among people without bathing. My conduct has now changed, I wash, bathe, comb my grey hair, look smart as I move among people. |  |  |
| Story 19 | Parenting skills |  | The change that has happened in my family is with my children. I told them from the start that I am studying because of them and they know it. I also always tell them that our teachers will not be happy if they come to visit us at home and find it dirty. So they clean because they know the teachers have ever come home and gave us soap to wash. This change is because of the intervention which enlightened us on loving and befriending our children, getting closer to them instead of barking and abusing them telling them you are not their mother, telling them not to return to your house if they go anywhere. Parents learnt how best to handle their children, now they don’t fear their parents and have also changed to having a better life. |  |  |
|  |  |  | The trick I use to talk to my children is that when I return home, I befriend them and buy them some sweets and later we start conversing as I bring in the relevant stories from what I studied when they are still happy and able to understand everything I tell them. When you people from the university came to our parish, you taught us very many things we dint know about and we studied and understood hence this brought about the change in our village |  |  |
|  |  |  | As, the elderly, while still young our parents raised us with an iron hand as a way of good upbringing. They would communicate to us with much force and emphasis. But during these study sessions, I realized that using force on a child may cause more harm than good. Thus handling issues with a child step by step can help the parent make them realize their mistakes and get them out of that issue at hand without having to first shout at them. The intervention study has taught us many tricks on how we can communicate with our children and when we talk, they listen to us and differentiate right from wrong. |  | Delineating the parenting skills of their caregiver |
|  |  |  | The trick that I use to talk to my children is calling and silently talking to them. For example, if she had gone away from home and returns late, I would call her aside and request her to explain why she returned home late and give her a chance to explain herself, instead of shouting and abusing her. |  | Listening to their children's perspectives |
|  | SRH communication |  | I have talked to my children about what we learned especially issues that emerge from engaging in sexual practices like getting unwanted pregnancies, diseases and death. I also talked to them about the stages they go through when maturing, body changes, challenges faced and how they can go about them, I always tell them about all this |  | Communication on SRH |
|  | Knowledge and attitude on SRH |  | As a learned parent, I didn’t know that children can also go for tests concerning STI’S and STD’s. But when I got informed, I talked to my children at home who were also not aware and were even scared of going to the hospital. But now they know about this. I also used to think that family planning is for us women as the radio adverts always say, “family planning is for women.” But through this intervention study, I got to know that children can also use family planning as a protection measure. |  | Learning that young adolescents can also be screened for STIs and that they can also be supported with contraception |
|  |  |  | I also learned that in case someone is raped, when rushed to the hospital there’s medicine that can be given to the victim as a way of preventing diseases and pregnancy which I really didn’t know is possible. |  |  |
| Story 20 | Parenting skills |  | As a parent, whenever I returned home, I never used to greet my children or ask them if all things at home are in place. Instead, if I found what I told them to do was not done, I would immediately shout at them. This intervention has first of all taught me that when I reach home, I should greet my children, asking them how the day was and if they were able to do their assignments without shouting at them so that the child is also given chance to explain what happened throughout the day. I now see the children becoming easier to handle and even report to you when they see something on their way; helping you know their movements. |  |  |
|  | SRH communication |  | You have to tell the child as a parent and I also have my 12years old daughter who has not yet experienced her periods, I told her that if she ever sees blood she should approach me, I talk to her and give her materials to use so that she doesn’t get embarrassed among other kids. I have discussed with my children on quite a number of things about what we studied for example my old girl who is already above 18 years, I tell her to avoid men as she would end up getting unwanted pregnancies. I also tell her to use pills in case she fails to control herself. I also teach my children to keep busy with work so that they do go loitering in villages and tell them to keep home if not sent anywhere. |  |  |
|  | Knowledge and attitude on SRH |  | What I didn’t know and learned is that 10 or 12 years’ child can get pregnant or get involved in sexual practices. I realized that this is actually the age where most children get spoilt. Therefore, I needed to sit my children down and openly tell them not to engage into sexual practices without getting shy. |  | Learning that 10 or 12 year olds can get pregnant |
| **Story 21** | Parenting skills |  | However, because of the study intervention, I had learned and obtained the experience on how to talk/communicate with my daughter without any fear, being man which had actually always been hard for me to do previously before I took part in the study. Whatever I advise her on, she stops it. This has helped very much in preventing her from the bad conduct that was developing and it is because I learned how to talk to her from the study sessions conducted. | Being able to talk to his daughter despite being a man |  |
|  |  |  | What has changed in my conduct is that whenever I would see a girl child in wrong, instead of talking directly to her about what she has done wrong directly, I instead would talk to the mother to discipline her but through the communication skills session, I gained the experience of talking to my daughter by myself in case of any wrong acts done. It is important to communicate with your children because if I don’t and my daughter ends up pregnant the community will put the blame on me saying if I the father and the mother had stopped that earlier, then she would get pregnant. Anything that happens to my | Emphasizes his role as being able to communicate with daughter |  |
|  |  |  | children is reflected on by the community, therefore I saw that it is very important to talk to my child as this will paint a good picture on the community who will say, how did Tindifa groomed his children that they are never involved in any bad acts which is very important to me. |  |  |
|  | Personal and Family life |  | I also communicate with my wife for example if I have 150,000 shillings, I call my wife and tell her that I have this amount of money which I earned through such a way, discuss with her on if this money can be saved on the bank account as school fees till the children return to school since there’s covid or we could invest it in purchasing a goat that could multiply by 30,000shillings by the time these children go back to school. This has also helped me very much because before I used to say, I earned the money myself and I can use it the way I want since I am the family head, without consulting my wife. If I got like 150,000, I used to buy 3kgs of meat for my family to also enjoy not knowing that my wife maybe could have a loan somewhere and could be paid using this same money and the meat waits until next Saturday but after the study sessions, I now engage my wife in planning for the money that I get so that incase she has something bigger than what I had to spend this money on, then we go for that first, and this study has helped me so much with doing this. | Being able to engage his wife in planning activities of the family |  |
|  | Community level changes |  | I can say that in our village there hasn’t been any change yet but we formed a group meeting with those who have been part of this study, agreed that if Julius’ son commits a crime, we can call him and solve the issue without having to go to court but this hasn’t been effective yet as the idea has just come up and just starting. | Groups were formed in the village for parents on how to support each other on parenting |  |
| **Story 22** | Parenting skills |  | What caught my attention the most is we were taught get closer to our children and become their friends and due to this, our children portray good behaviors. Before the intervention our children would abuse people but now when u guide them not to do that, they have stopped. |  |  |
|  | SRH communication |  | We learned how to communicate with a girl child who has started their puberty stage and how this child should communicate with you for example when the girl child has stated to experience their menstrual periods and they tell you, as a parent you should advise them that their blood shouldn’t be seen and when my daughter told me I got for her all the necessities, bought for her pads, nickers and soap. I also told her that when a girl starts menstruating and engage in sexual act, they get pregnant therefore she shouldn’t do that. |  |  |
|  |  |  | In my generation when a girl would start her menstruation periods and this is how I raised my first children who are all girls, you would call the child, make her sit on the fathers bed and make her face up on the roof as a way of reducing the blood flow and then cut for her a clean cloth to put on her pantie so that the blood doesn’t pass through. Also during this time, I would guide my children and tell them not to engage in sexual acts because they would end up pregnant which would bring them issues as they would isolate themselves from the public and not behave the right way. I would tell them to keep home, protect themselves and wait for the right time when they grow and finally get married. |  | Communication on SRH coupled with cultural practices |
|  | Knowledge and attitude on SRH |  | We also learned how to communicate with a boy child in puberty, his voice changes, pimples develop on his face as a sign that he has now matured and is no longer a child so we advise them not to go along with girls because he can easily impregnate them and that if he engages himself in sexual acts, he will acquire | Sexuality communication can take place between a mother and her son. |  |
|  |  |  | HIV/AIDS which will also happen to the girl child hence advising him not to engage in sexual acts before maturing fully as we were taught. |  |  |
|  | Personal and Family life |  | The change that is in our village is that the intervention study added on where we were for example they taught us to love ourselves, and said as a woman you should know your husband and also know yourself therefore you shouldn’t move anyhow but instead protect yourself and no man will love a woman who just moves aimlessly. This has added on what we knew as people. |  |  |
|  | Intervention Delivery |  | What helped me to learn well from the study sessions we had was the role plays that were conducted for example there’s a role play where a girl child got pregnant but feared to tell her parent so she didn’t end up well. Another one was a child who had a burden of very many problems, we put stones in a sac which she carried but she got tired and put the sack down because she was all alone, we all started picking stones from the sack and this helped this girl child so feel some relief and she was happy and got back on track. What I learned from this is that we all have many problems but don’t have who to tell but when we meet some people and talk to the about what we are going through, they console us and make us feel much better. | The role plays used in the intervention |  |
| **Story 23** | Parenting skills |  |  |  |  |
|  |  |  | What changed on me is that I added on what I knew and got better. I also didn’t know how to handle children easily because I have some of them who are now married but whenever they did wrong, I used to shout at them and also beat them up because I am a short tempered person but now when my children go wrong, I call them talk to them and guide them on what to do without being rough and this change is because of the study intervention. | Abandoning old parenting styles and adopting new ones |  |
|  | SRH communication |  | My boy is 14years old and my girl is 2 years below him. I called my boy and told him that he is now 14 years and has now grown and should concentrate on his studies, if he messes up and sleep with some else’s daughter and impregnates her and for him he expects to keep in school, it won’t work. I also asked my daughter if they have taught about menstruation at her school or if the mother has to her about it and she said she knows about it. | SRH communication with son and daughter, with daughter it is about menstruation with son it is about the consequences of getting the girl pregnant |  |
|  |  |  | . I told her that if her menstrual periods begin she should tell either me or the mother so that we can get for her what to use. I also asked the younger sister and after that I told them that there are many bad things that originate from practicing sexual intercourse, have you heard about them and the answered that they know and have heard that on the radio that you can get diseases and suffer from HIV/AIDS. They also told me that there’s someone from the university who came at their school and taught them about that. Then I advised them to do all they can to protect themselves from such acts and told them that if they ever chase them away from school because of school fees or anything that they didn’t have, they should come home immediately not first roaming around. |  |  |
|  | Knowledge and attitude on SRH |  | We learned so many things, we started by being asked who a parent is which we explained, learned the role of a parent. We later learned about child development and how parents should take care of their children, learned about puberty and the stages that both boys and girls go through while in puberty for example a girl starts to menstruate at an age of 10 years and above, even boys start to experience puberty at this same age yet I personal thought this happens when the child is 18 years. We also learned how a parent can handle a child who is in puberty so that they can understand them, learned who a good parent and a bad parent is, learned about relationships and how to guide our children in making health relationships, encouraging them not to look for bad friends. We also learned to prevent our children from engaging in sexual acts by telling them that there is disease like HIV/AIDS and STIs and that’s when they explained the meaning of ABC. | Narrative on what was taught and learned during the intervention |  |
|  | Community level changes |  | I have a neighbor whose children used to be moving around aimlessly and after studying I called him and told him to give his children some work to do, to always go with them to the plantation when going to dig and now these children no longer roam around. | Building community support systems for parenting |  |
| **Story 24** | Parenting skills |  | The reason why there’s change is because of the study intervention because before it happened, I didn’t know any of the things they taught for example; a child is like a seed, he/she needs to be watered, pruned in case they develop many leaves and we saw that even if a child annoys you forgive and guide them. Also when your neighbors’ child falls in trouble don’t laugh at them but instead talk to them about everything even if they have gone through it, counsel them for as long as you took part in this study intervention. Thank you very much. | Learning how to nurture a child |  |
|  | SRH communication |  | What I put in practice is guiding my 10-year-old boy to be careful. I told him that he is going to experience some body changes during this period and that he would also experience dreams sleeping with girls and also develop that urge but openly told him that at his age if befriended a girl and sleeps with her, the gal would get pregnant and that he would also suffer from diseases like HIV/AIDS so he should engage himself in such act. I also told him about ways on how he can protect himself. | SRH communication especially on topics deemed sensitive like wet dreams |  |
|  |  |  | .At first he used to fear me a lot but now I see he doesn’t, when he encounters a problem or wants something he comes tells me directly for example there’s a time he came asking me what HIV/AIDs is, and I told him that it is a disease that spreads through sexual intercourse. He asked me what a sexual intercourse is and I explained to him that it is when a boy and a girl engage in having sex and I told him that at his age if he practices such acts he would suffer from HIV/AIDS or impregnate the girl and I also told him what happens when someone suffers from this disease which he understood. | Building a relationship with a child has allowed them to be able to discuss SRH without any difficulties |  |
|  | Knowledge and attitude on SRH |  | I learned how to communicate with my child especially at the age of puberty. I also learned about the categories of parents which included either 4 or 5 kinds including; the caring parent, strict parent and one who doesn’t mind. I also never knew that it was important to talk with my child who is in puberty because I knew when a child reaches that stage then know what to do and need no guidance but I learned how to handle such a child, call them and talk directly without fearing or hiding anything. I was taught that even it is a boy and you are a woman you can guide the and even if it is a girl and the father is around, you can still talk to them. We were also taught the tricks we can use to talk with these children. |  |  |
| **Story 25** | Parenting skills |  | The topic that became very important and brought change in my life is when we looked types of family heads and leadership in homes which included the caring parent, the care free parent and the strict parent. After the study session I evaluated myself and fell in the category of the care free parent because I really had no time for my children, even when they would ask me for something, I would just buy it without assessing why they needed it therefore I picked a lot of interest in this topic because I realized I had lost a lot from my conduct which made me a changed person and I now started to give my children time and getting closer to them. I have a grand son and a granddaughter and other old children that I have now got close to and they have opened up with me, and I was lucky that they tell me every challenge they face even what I have not asked for I am able to know, we converse together as though we are age mates, agree and I give them guidance on how to overcome the challenges they face especially my grandson and I see that things have become better especially during this covid period when we have stayed a lot with our children because now my children have become very close to me and share with my there problems and I guide them. |  |  |
|  |  |  | his intervention has contributed the most on the change that has happened. Just like I told you I used to be a care free parent but I am now a changed person because when I reached here, they taught us about growing a plant I realized that I was missing a lot on nurturing my children like that plant. Other things that have brought some change are listening to the radio and engaging with friends. | Transformation from a neglectful parent |  |
|  | SRH communication |  | My grandson is very funny, he sometimes tells me that there’s a girl who is always on stuck on him, though schools closed due to covid there are always teachers that teach from homes and because this boy is clever they like him and invited him to study so that he doesn’t miss out a lot but of course they made me pay some money. It was on Friday when he told me about this story, as he was coming back from studying, there’s a girl he came with and while on the way the girl told him that they should play hide and seek and whoever finds the other will do whatever they want to them. Because it was getting late and my son need to be home before it got dark he refused to play with that girl and said they would play next time. When he told me this story, I told him playing is okay but sometimes we have to use our brains and I asked so what were you going to do if you found the girl and she told you to do anything to her, he said he would wrestle with her and I asked what would you do next, he said nothing. I used this chance to openly tell him because it is always good to tell you children things the way they are, call a spade a spade because there’s a neighbor who cut his friends penis and we rushed him to the hospital because he saw the father cutting the cow’s tail and was calling it a tail so when the boy got home he cut the friend’s penis calling it the tail of the cow. I told him that he is now old enough and when boys get feelings for a girl, the penis stands because he is attracted to her and if he | Parent child communication and more explicit but sometimes marred by threats |  |
|  |  |  | tries to have sex with her the penis will end up bleeding since he has never experienced such a thing, he will suffer from diseases, can be taken to prison or be beaten for no good reason. He said that he is young and getting diseases how, I explained to him that diseases will get anyone they don’t look for age or color or size and also told him that incase he impregnates someone’s daughter, he will get imprisoned and lose out on school, I know how much he loves to study. |  |  |
|  |  |  | Also my wife has changed because she is a person who is short tempered and whenever she came home and found something is wrong, she would start shouting and beating the children without first finding out who did wrong, I talked to her about her reaction told her that beating the child because they broke a cup won’t bring it back, shouting at a child when in wrong is also not good and I see she has now changed, she tries to first know wat happened before reacting and this made me happy and I have also changed to better, I’m no longer the care free parent I used to be. |  |  |
|  | Community level changes |  | To sum it all, the intervention helped my children and also when I’m talking to my friends, I tell them about what I studied, some of them agree to it and other reject it. I am a person who likes sharing and I have a talent of public speaking, I’m always an MC at parties but now I refuse because I have aged however when I’m invited at a burial, there’s no way I can refuse and whenever I’m talking I bring out such stories on what we have been studying, there are some people who listen pick something and change their ways but of course there are those who are big headed and refuse to listen however learning is a gradual process so we keep preaching just like in church, they keep preaching and don’t stop and a few keep changing. |  |  |
|  | Intervention Delivery |  | The method that helped me grasp well what we studied is that I used to write notes and also we studied some things practically for example we made a role play where some parents attacked the child while from school and us when the child return from school we called her, talked to her and advised her on where she had gone wrong. Another thing is that we had good teachers. | Role plays by the intervention |  |
| Story 26 | Parenting skills |  | Also the study intervention united us parents in a way that when I have a child that I have failed to handle or tried to guide and cannot explain because they fear me I take them to another parent who took part in the study and they help me with that child and when that parent also has a child that they have failed to handle, I can help with that because there is when a child fears you even though you are the parent and cannot tell you anything. | seeking parenting support. |  |
|  | SRH communication |  | I have forgotten the rest but it helped us a lot and we are now no longer shy when teaching our children about religion, knowing God, teaching them how to pray because we used to threaten the girl child that is got pregnant through carelessness, she would eat the baby. But now after the study we realized that when you sit down with a child and talk to them they understand you better and they would know that where they are going is not safe so they have to protect themselves. First of all she would get pregnant, suffer from diseases that originate from sexual practices e.g. HIV, candida gonorrhea but when you have told the child to protect themselves when they go or to be patient, study until they find their partners and they if they fail to follow this advice they should use family planning. | Attaining good communication skills on SRH from using threats to having discussions with the children |  |
|  | Knowledge and attitude on SRH |  | I was excited because learning doesn’t stop even though we are aged; it is necessary to study. I liked it because our youth has the way they treat us but after teaching us we gained the morale of disciplining, guiding and communicating with them because at first we were shy to talk to them for example talking to our children on how to conduct themselves but we got the courage to be free with them in conversing, consoling and telling them how to conduct themselves. As parents it helped us because we used to think telling my child something like using family planning or using a condom will ashamed me. But basing on what we studied we should tell the children when we are free and when the child has a problem they also tell me and I explain and console them. | At first there was shyness regarding discussion of some topics such as family planning or condoms thinking that it was embarrassing. But the sessions have helped them to talk more freely about these topics |  |
|  |  |  | We studied about hygiene in women which is in different ways like at home, bedroom and to be clean as married women in bed. There is also pulling which was known as visiting the bush but now people use soap to bath and clean their genitals and go but you find there is something when having intercourse with your partner and things don’t go well but when you have visited the bush, there’s no smell at all and everything goes well and this taught us as women to be clean. Children of this generation however don’t want to be guided by their aunties who tell them that being hygienic helped us and it is not good to smell when with their husbands because they won’t love them, even if they listen, they will not put in practice what they were told to do. The intervention also helped us to be free without children and sit with them on table to get their thoughts. | Persistent t cultural attributes like labial elongation which are also a misrepresentation of what was taught |  |
|  | Personal and Family life |  | We also learnt how to take care of our families, cooking food, taking care of the young ones and the visitors who come at home. They taught us how to save, | Entrepreneurship skills to enable them support their |  |
|  |  |  | that we are supposed to plant ,sell some and save the surplus | adolescents with some needs like provision of sanitary pads |  |
|  |  |  | What I learned from the study sessions children fear their parent and help them, I learned to save for example if I harvest 2 sacks of beans I should sell 1 and save the other, financial literacy because some people have been keeping their money under their pillows | Financial literacy |  |
|  |  |  | I taught my children to forgive each other in case one annoys the other because they always keep anger and I hear them fighting and quarrelling with each other but I told them that if someone disturbs annoys you, tell me and we sit down together and solve if so that you keep at peace with one another and always forgive each other because I want you to be good people with good hearts. Sometimes we say that we are tired of our children but as parents God gave us a responsibility of taking care of our children for as long as we are still alive, if you don’t have children take care of the grandchildren, guide them and if the go then you can guide your great grandchildren. I learned a lot and sometimes I go to my note book and read so much that I didn’t know about because there are questions that were asked and I find I didn’t know them therefore this intervention brought light to us parents. | Ensuring harmony among the children |  |
| Story 27 | Parenting skills |  | The things that we study that brought change in my life is one we started with studying that a child is like a plant and for a plant to grow well there a certain things one need to do therefore we learnt that you should care for the children and give the time to understand them better and this helped me because I used this technique on my child | Nurturing a child |  |
|  |  |  | My boy is 12 years old and I see that he has changed positively because he now knows that boys can also peel, peeling is not for just girls, a boy can also mob the house | Socializing boys-that can take on some roles traditionally meant for girls |  |
|  | SRH communication |  | and I also warned him about girls. I told him that if a girl that is older than him ever asks to sleep with him, he should know that he will suffer from HIV/AIDS and even die before he grows up because that act is not good for him at his age. He also told me that he now fears girls because of what I told him about them and I have noticed that change. | SRH communication still marred by threats |  |
|  | Knowledge and attitude on SRH |  | There are insights that you taught about that I didn’t know for example a 9 years old girls starting to menstruate or getting pregnant, we didn’t know that this is possible, we knew that this can only happen when the girl reaches 15 years, in fact we all failed the question that was asked in the paper asking if it is possible for a girl child of 10years can get pregnant. | learning that a 9 year old can also get pregnant |  |
| **Story 28** | Parenting skills |  | first I saw no reason for a child and a parent to have a talk but after this intervention my children have now become my friends. Before a child used to fear their parents and even when they encountered a problem, the would never say it and also when the parent wanted to tell something to a child, they found difficulty in telling their children thinking it is bad. But after this intervention, my children have become my friends, we sit down and converse and even they come here like now, you can see the change and I thank you very much for this. I talk to them about the challenges the could face at their each if the engage themselves in certain acts and they also update me in what things they encounter during and I use this time advise them on what is wrong and what is right. |  |  |
|  | SRH communication |  | Specifically, the boys that are 14 years are adolescent and me personally I would never sit down with them to tell them things like if you come across a girl and sleep with them, you will end up suffering from HIV/AIDS and other STIs or even impregnate someone’s daughter which might turn out a problem, you might get out of school, disappear from home and even for us your parents we shall be blamed for your mistake and also they come telling you what they met wherever they were during the day. I also tell them that a young boy should be returning home early because moving in the night will lead him into bad peer groups so sometimes he tells me that he isn’t going to the pitch today and this makes me feel I’m now closer to my children. |  |  |
|  |  |  | Talking to my children has become very important for me, because they are now open enough with me, one of them came and told me that his penis hurts whenever he is urinating and I took him to the hospital yet this had never happened with my children before, they never used to tell me anything about what is happening in their lives. | Children can now comfortably approach parents on their issues |  |
|  |  |  | What has brought change in my love mostly is loving my children and it has become something very big in my life. I really didn’t have time for children before even though I used to bring them some items from wherever I would be but now my children are my friends and that is something very big that was not in my life but now is there. Secondly, during the study intervention, we were taught to love our husbands and care for them because the appearance of a man in public reflects on the home/family he comes from. If a man is wearing shabby clothes in public, that means that that how the wife is and after the intervention I started to take care of my husband in that when you meet him somewhere, you can at least say that this man has a caring wife, and now he also says it that he is proud of me. I learned this also and it has become important in my life. Another thing is that I learned to organize my home so that when someone comes around they can at least testify that I am a learned woman. Myself when I am coming from church I look at my home and admire it saying it is really good to be a learned person and sometimes before I leave home, I first ask myself that what if I leave the home disorganized and our teachers come to visit, how will they find the home surely and then I say now even if anyone comes it is okay, I can be conversing with them with serving them tea. All these things have become very important in my life. |  |  |
|  |  |  | The study intervention has the biggest change in my life because even before you came, we had always moved but I had never got any close to my children and to tell you the truth, I adopted this from only you after this intervention. |  |  |
|  |  |  | At the start we studied very many things and this was not just about the children, we also learned to educate our children because some parents were not caring and would luck money saying whatever happens is fine, but after this study intervention, I realized that if you don’t educate your child, you are working for no good reason therefore I promised myself that I must use my two hands and do whatever it takes to make sure that my children keep in school, never lack books or school fees so that they can reach the level of education I was not able to reach since I am not educated enough but I feel my children must achieve what I failed to achieve and I also thank you very much for this, may God bless you. |  |  |
| Story 29 | Parenting skills |  | The change that has happened especially for us with children, both boys and girls is that I never used to have conversations with my children but now I give them time to talk to me. Before the intervention I used to say that children belong to the mother and she is the one to listen to all their problems but there is when they don’t tell her so I have to give the time and listen to them. | Changes in gender perspectives |  |
|  |  |  | The challenge that I have encountered as I communicate with my children is that sometimes you talk to children but not all of them will listen to you, one will obey and the other will not but still this intervention has taught us not to be hard on the children, rather handle them in a simpler way, personally I don’t even recall when I last beat my child, I instead punish them with words. |  |  |
|  | SRH communication |  | A lot of change has occurred within our children during this Covi-19 period, I have children who are both old and those still young and I observed that during this covid, they have started to move up and down so I sometimes call and talk to them, asking them why they be loitering, what they be looking for and also warn them that they will most likely come along bad things as the loiter. A child must be told that they have now grown and they need to be more careful, for example I warned my boy that he shouldn’t move around with people’s daughters and that if I ever hear or meet him with a girl, we would fight. After this he changed and only goes to play football which I can’t restrict them from doing however I always follow up asking, where he played from, which team he played with… which I never used to do. |  |  |
|  | Knowledge and attitude on SRH |  | We have always done some of the thing but the intervention taught us things many things we didn’t know for example changes in adolescents. I have a child who is developing breasts and I have talked to him to fear that, I advised him to always press them so that the liquid comes out because if he fears the pain the breasts will develop and also told him about the myths in the past says that when a man grows breasts and they come in contact of the woman’s breasts, the woman will die and this means that this boy will not be able to marry. I see my boy is now trying to work this out. | Knowledge in demystifying some misconceptions |  |
|  | Parenting skills |  | I used to be very strict and tough on my children which made them fear me and never used to tell me anything but now I am close to them and they are my friends, we converse and they tell me what they are going through. Of course children can’t tell you everything but I see that we talk about most, converse and share thoughts. It’s no longer hard for me to talk to them and also no longer hard for them to talk to me and I see we talk a lot with my daughter who is 14 years old. This has not only helped this 14 years old girl but even the old ones of about 16years and the young ones have benefited because talking to your child would be really hard, we had no time, wake up early morning direct them on what to do and go to the garden to weed but now you have to make sure you talk to them, maybe during lunch time, or the child comes herself and finds you in the garden and tell you what the problem is because they are now friends with you. |  |  |
| story 30 | SRH communication |  | My girls who are 14 years had not started their menstruation periods and when it happened they approached me saying mum, we have started our periods so we need pads which was not done by their elder sister, even the young boy came telling me that his pant is old which is a sign that for these young children I have treated them in a different way, they are my friends and are free to tell me anything which wasn’t the case with my older children. |  |  |
|  |  |  | My daughters reported to me that my son was getting closer to some woman and that he was going to get spoilt, I used this trick on my boy and told him that never get used to old women, u can sit with them listen to stories and talk buy if she ever asks you to sleep with her, you will become a nuisance, no knowledge will be left in your mind and you will become very useless never to do anything important for yourself because you slept with and also woman, I told them that old women are witches but warned them not to meet people on the way and start calling them witches. I added many more words so that I can scare them so as they are able to do the right thing and of course I know that they will make research and find out that I lied to them but by then they will have already grown old. | SRH communication on cross generational romantic relationships |  |
|  |  |  | My thoughts about children engaging in sexual acts changed after attending that session about STDs and HIV. My children had not started engaging in those acts but I took time and talked to them about this, I asked them if they knew about AIDs and if they know that it kills and one of my children said she had cut herself and asked if that meant she was going to suffer from HIV/AIDs. I told her that it is possible to get AIDs through cuts but the most important thing right now is to protect themselves from men especially now that they have already started menstruating because they can become pregnant and also suffer from HIV/AIDS. Some times when we are weeding in the garden, I tell them that do you know that this person almost died, and they asked me of the cause which I said was AIDs. I also told them that condoms can prevent one from getting pregnant, I talk to them freely because we are now used to each other and the ask me questions basing on what they know from school which was not the case in the past because I used to fear telling them about such a thing but the more they ask me the more I use the chance to explain to them because when they ask questions then you get a lot to talk about and they can only ask you questions when they don’t fear you. |  |  |
|  |  |  | Talking to my children and having a good relationship with them is very important to me and has changed my life because having someone that is open with you makes it easy for both of you to understand each other and this was a good move to take care of my children because if I wasn’t open with them, they also wouldn’t be open with me and also your friend is that one who is open with you and tell you everything. If at all you have a friend that is not open with you and you find out that they don’t tell you some things, then you lose your trust in them because your friend is one you approach when you have a challenge and reach out to and this was an important aspect for be to build a good relationship with my children. |  |  |
|  |  |  | My children started their periods when they were 14years old, when one of them told me that she had seen blood on her knickers when she woke up in the morning, I asked her if she sleeps in the knickers and I told her yes. I the discussed with her and told her that when we were still young we used a piece of cloth but for them they will use pads. I also told them that if they ever go in their periods while I am not around or incase the money I gave them to use is over (they were already boarding at school in P.6 before covid) or they are somewhere where there are no pads, they can use a piece of cloth or a hankie, fold it well and put it on and wear a tight knickers so that it doesn’t fall. I also told them to tell me if their knickers become loose so that i buy for them others, told them that the cloth has to be changed and washed because it smells a lot, not like the pads that might not smell. I also taught them that they have to use a razor blade to shave before their periods start and that they have to always bath in the morning, in the afternoon and in the evening because during this period girls tend to smell so they have to keep very clean all the time, that’s what I tried to talk to them about. |  |  |
|  | Knowledge and attitude on SRH |  | In most cases parents run after girl children and ignore the boys yet the boys can also get spoilt, become big headed, impregnate other girls and most women here have children yet they are still young because they are impregnated by boys in their age groups who have to study hence living the burden to their parents which is really a big mess. Another issue that can arise is that boy moving around with old women where they learn bad habits, in my home I talk about it, but as always said only God can change some one’s heart however we should try our best and God works within our efforts. I grew up knowing that when you scare a child about the wrong they do when they are still young, that child will grow up when they fear to do that act. Also when you were teaching us about what life was like in the past, what is in the present and how it will be in the future for example parents would groom their children by telling them that if the sit on cooking stones, then they will get burnt. I also tried this trick and told my children that if you don’t wash utensils then you will become old quickly so whenever utensils are not washed and I tell them that they run to wash the utensils. | Change in attiude toward s boychild and the fact that they need as much attention as the girls |  |
|  |  |  |  |  |  |
