# Supplementary material for: Narratives of most significant change to explore experiences of caregivers in a caregiver-young adolescent sexual and reproductive health communication intervention in rural south-western Uganda
Source: PLoS One. 2023 May 31;18(5):e0286319. doi: 10.1371/journal.pone.0286319 (PMC10231775; doi:10.1371/journal.pone.0286319)
Supplement: Attachment — Submitted filename: Reviewer Report.docx [file pone.0286319.s003.docx]

**Reviewer Report:**

I am writing this report as a reviewer of the manuscript entitled *“Narratives of most significant change to explore experiences of caregivers in a caregiver-young adolescent sexual and reproductive health communication intervention in rural southwestern Uganda”*.

Using most significant change stories, the authors have tried to explore the experience of SRH communication intervention among the caregivers of young adolescents. The study found several interesting findings like improvements in communication between the caregivers and young adolescents, improvement in positive parenting skills, increased harmony between the caregivers and YAs, changing attitude towards gender roles among the caregivers who attended the training interventions.

I would suggest couple of recommendations/suggestion for further improvement of this paper. However, authors can use their discretion to agree or disagree with the feedback and consider the revision.

**General comments**

1. YA, YA children, children and adolescents are being used interchangeably throughout the manuscript. I would suggest making consistency.
2. I was interested to know what the interventions were. Although table 1 presents the outline of the intervention, I was interested to know more about the intervention.
3. Training modules were delivered through small group sessions (10-20 participants) for 15 weeks, I was interested to know how many days in a week? How many hours in each session? What was the teaching learning approach? How was the review done? Thus, I would suggest presenting more detail of intervention somewhere in the supplementary files so that it would be beneficial for those who are implementing similar programs.
4. The authors need to justify the number of stories they collected, and they included for the analysis. There are some possible biases that might have occurred while conducting this study. How has the authors addressed the bias?
I. Possible bias after selecting the participant purposively and selecting all the stories that were collected,
II. Although the authors have mentioned about their limitation in the discussion section, I was interested how the participants/respondents were identified by the researcher who was an external
III. Selecting the researchers for collecting the stories (Possible bias towards the views/text from the researchers who are good at story telling
5. Make consistency in using active/passive voices throughout the paragraph, (Example Line 181-183 or tense (Example; Line 187-188), line 253 or punctuation or spelling (example line 233, 153).
6. The manuscript needs paraphrasing and making simple sentences in several places to make the readers understand easily.
7. Check the referencing and citation style for this Journal and revise it accordingly

**Specific Comments**

| **Section** | **Line #** | **Comments/Feedback** |
| --- | --- | --- |
| **Abstract** |  | There is unclarity between background, methods and findings. The authors need to clarify this |
| **Background** | *46* | What do the authors mean to mention by YA here? It would be better if there is definition of YA in the introductory paragraph. Although the authors have defined YA as children aged 10-14 years in line # 92, it would better to be mentioned here in the introductory paragraph. |
|  | *49-52* | Can make two sentences for more clarity to the readers |
|  | *52, 69* | Full form of CSE, Use abbreviation in line #69 |
|  | *74* | What do you mean by quality of relationship here? |
|  |  | What is the rationale for using MSC as a technique for this study? |
|  |  |  |
| **Methods** | *105* | Mean age of caregivers should be in findings rather than the method |
|  | *107* | Is it the population estimate for 2022 or the findings of census data of 2019? |
|  | *114* | How the guidelines were culturally sensitive? What culturally sensitive issues do those guidelines mean to address? |
|  | *144* | Is there any rationale for using MSC technique for evaluating the intervention? |
|  | *173* | Who were included in the plenary focus group for verifying the stories? |
|  | *194-195* | How was the privacy and confidentiality maintained during the data collection process? What was the setting of the interview? |
|  |  | Was the data analysis done manually or was done by using software? |
| **Results** |  | Socio-demographic profile of respondents is required here. Please move the age-related information from methods to result section. |
|  | *366* | The authors reported changing attitude towards traditional gender roles of children (372-374). What was the intervention that the caregivers did with there children to change such gender norms? Was it observed in more than one stories? |
|  |  | I suggest the authors to present the domains of change in a figure somewhere in the result section so that the readers will easily understand |
| **Discussion** |  | Since, the findings are based on the predetermined domains of change (line 166), triangulating the findings with quantitative findings could have been better. Although the authors have triangulated the findings from quantitative study (Reference #26), more discussion of such findings and other quantitative programmatic data might be required |
| **References** |  | Some of the references are incorrect or incomplete (Reference #9). Please check all the references are correctly cited. |
